# Exogenous Ascorbic Acid Induced Chilling Tolerance in Tomato Plants Through Modulating Metabolism, Osmolytes, Antioxidants, and Transcriptional Regulation of Catalase and Heat Shock Proteins

**DOI:** 10.3390/plants9040431

**Published:** 2020-04-01

**Authors:** Amr Elkelish, Sameer H. Qari, Yasser S. A. Mazrou, Khaled A. A. Abdelaal, Yaser M. Hafez, Abdelghafar M. Abu-Elsaoud, Gaber El-Saber Batiha, Mohamed A. El-Esawi, Nihal El Nahhas

**Affiliations:** 1 Botany Department, Faculty of Science, Suez Canal University, Ismailia 41522, Egypt; amr.elkelish@science.suez.edu.eg; 2 Biology Department, Aliumum University College, Umm Al-Qura University, Mecca 21955, Saudi Arabia; shqari@uqu.edu.sa; 3 Business Administration Department, Community college, King Khalid University, Guraiger, Abha 62529, Saudi Arabia; ymazrou@kku.edu.sa; 4 Faculty of Agriculture, Tanta University, Tanta 31512, Egypt; 5 EPCRS Excellence Center, Plant Pathology and Biotechnology Laboratory, Agricultural Botany Department, Faculty of Agriculture, Kafrelsheikh University, Kafr El-Sheikh 33516, Egypt; Khaled__elhaies@agr.kfs.edu.eg (K.A.A.A.); Yasser.abdelgawad@agr.kfs.edu.eg (Y.M.H.); 6 Department of Pharmacology and Therapeutics, Faculty of Veterinary Medicine, Damanhour University, Damanhour 22511, AlBeheira, Egypt; dr_gaber_batiha@vetmed.dmu.edu.eg; 7 Botany Department, Faculty of Science, Tanta University, Tanta 31512, Egypt; mohamed.elesawi@science.tanta.edu.eg; 8 Department of Botany and Microbiology, Faculty of Science, Alexandria University, Moharram baik 21515, Alexandria, Egypt; nihal.elnahhas@alexu.edu.eg

**Keywords:** Ascorbic Acid, chilling, tomato, gene expression, catalase, Heat Shock Proteins

## Abstract

Chilling, a sort of cold stress, is a typical abiotic ecological stress that impacts the development as well as the growth of crops. The present study was carried to investigate the role of ascorbic acid root priming in enhancing tolerance of tomato seedlings against acute chilling stress. The treatments included untreated control, ascorbic acid-treated plants (AsA; 0.5 mM), acute chilling-stressed plants (4 °C), and chilling stressed seedlings treated by ascorbic acid. Exposure to acute chilling stress reduced growth in terms of length, fresh and dry biomass, pigment synthesis, and photosynthesis. AsA was effective in mitigating the injurious effects of chilling stress to significant levels when supplied at 0.5 mM concentrations. AsA priming reduced the chilling mediated oxidative damage by lowering the electrolyte leakage, lipid peroxidation, and hydrogen peroxide. Moreover, up regulating the activity of enzymatic components of the antioxidant system. Further, 0.5 mM AsA proved beneficial in enhancing ions uptake in normal and chilling stressed seedlings. At the gene expression level, AsA significantly lowered the expression level of CAT and heat shock protein genes. Therefore, we theorize that the implementation of exogenous AsA treatment reduced the negative effects of severe chilling stress on tomato.

## 1. Introduction

Global warming represents one of the most significant research concerns currently confronted by biologists and agronomists [1]. Plants were grown under open environments often pass through intervals of abiotic stress throughout their lifetime [2]. Previous studies recorded the adaptable responses of crop plants to various abiotic stresses, in which focus has been laid on particular stress factors [3]. Based on the Food and Agriculture Organization (FAO), the human population is growing fast and may reach around 10 billion in 2050. Additionally, food production is lowering due to different abiotic stresses. Consequently, the earth will need 70% more food by 2050 [4,5]. Thus, it is essential to acquire crops that exhibit equally improved vigor and tolerance to different environmental factors to face the upcoming challenge of food security [6].

Chilling stress may appear within temperatures among 0 and 15 °C [7,8]. Chilling stress causes numerous phenotypic signs, such as reductions in photosynthetic pigments, chlorosis, wilting, and cell death [9]. Furthermore, various metabolic modifications are triggered as a result of chilling stress, such as decreases in unsaturated fatty acid contents and increases in the permeability of cell membranes, which collectively reduce plant efficiency [8]. Subsequently, plants undergo different mechanisms for adaptation and protection in low-temperature stress. This maintenance mechanism simplifies the decreases in the efficiency of photosystem II (PSII), modifies the carbon reduction cycle and photosynthetic complex systems and speeds up the formation of reactive oxygen species (ROS) [10]. Moreover, it activates downstream signaling approaches for transcriptional regulation [11]. Up-regulation of mitogen-activated protein kinase transduction pathway [12], ROS signaling, and heat shock protein (HSP)/signaling pathways seem to be the leading players in the transcriptional regulation of plant under chilling/heat stress [12]. HSPs comprise a stress-responsive family of proteins whose were initially recognized when they accrued in plants as a result of heat stress, and then on it was found that they play essential functions in thermotolerance [13]. Several environmental and developmental conditions found to induce accumulation of HSPs, such as fruit ripening [14], low temperature [15], and oxidative stress [16,17]. Furthermore, the deposition of HSPs usually not just confers defense as opposed to the stress that triggers its synthesis, it even towards all upcoming nerve-racking scenario [18].

However, transgenic or breeding strategies revealed considerable outcomes in improving stress tolerance in plants [19]. Nevertheless, it cannot be effectively accepted to the agronomical community. Thus, extensive attention has been focused on lowering the deleterious effect of chilling stress through the exogenic implementation of various priming forms [20,21]. The usage of several compounds like sitosterol, magnesium, and potassium nitrate ready boost plant stress tolerance [11,22]. As a result of urbanization and the reduction in water resources and farmland, modification in the growth medium is a choice for sustainability [23]. The recognition of hydroponics strategies is increasing because of its excellent results in saving resources and improving food production [24]. Most plants might be grown using hydroponics such as leafy vegetables and tomatoes [25]. Root priming is one of the recent and efficient priming strategies, and the priming compounds are directly employed in the root region and stimulate abiotic stress tolerance. Root priming is also a popular research practice due to the direct exposure to the tested priming agent to evaluate its further consequences on the plant.

Ascorbic acid (AsA) is among the valuable metabolites associated with cell division, and osmotic adjustment. Vitamin C additionally possesses powerful antioxidant potential and assists in controlling the formation and scavenging of ROS [26]. Remarkably, the exogenous implementation of AsA might boost the endogenous AsA level [27]. Within the current study, we assumed that 0.5 mM ascorbic acid might trigger systemic tolerance in tomato under low-temperature stress (4 °C, 7 h). Hence, the impact of the root priming with ascorbic acid were evaluated on the biochemical, and molecular level.

## 2. Results

### 2.1. Phenotypic Remarks

**Figure 1:**
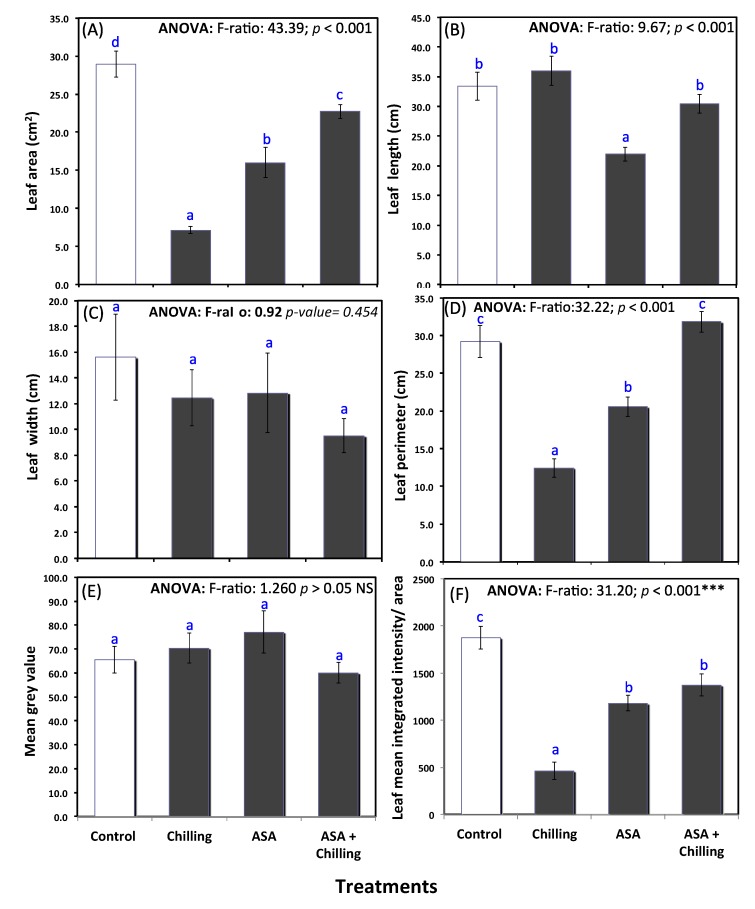
Phenotypic measurements of tomato plants under chilling stress and pretreated with ascorbic acid (0.5 mM); (**A**) leaf area, (**B**) leaf length, (**C**) leaf width, (**D**) leaf perimeter, (**E**) mean grey value, and (**F**) leaf mean integrated intensity/area. Different lowercase letters indicate significant different according to one-way ANOVA and Duncan’s multiple range tests at *p* < 0.05.

The subjection of tomato to chilling stress leads to various morphological and phenotypic deformation, including leaf curling, wilting, and shrinkage of leaf blade (Figure 1). On the other hand, plants pretreated with exogenous ascorbic acid (0.5 mM AsA) showed an obvious alleviated effect against chilling-induced stress, as supported by the leaf phenology, turgor, and structure (Figure 1). Tomato plants treated with exogenous AsA and exposed to chilling stress displayed similar phenotypes with control plants (Figure 1), confirming that AsA has a potential role in alleviating the chilling stress damage symptoms in tomato plants.

Before exposing the plants to chilling stress, tomato plants showed an average shoot, root, and plant fresh weight of 3.57, 1.32, and 4.90 g FW^−1^ Plant, respectively (Table 1), with the major part of biomass allocated towards the shoot system (about 72% of biomass). The lengths of the shoot, root, and plant measured were 12.86, 11.81, and 24.67 cm, respectively (Table 1).

The tomato plant leaf area decreased significantly after exposure to chilling (28.94 cm^2^ in control group compared to 7.16 cm^2^ in stressed plants). However, plants treated with exogenous AsA showed leaf area compared to control (22.76 cm^2^; Figure 1A). Leaf length and width showed no significant difference among all treatments, including the control group (Figure 1B,C). Leaf perimeter showed a similar trend as by leaf area; no significant difference between control and plants treated with AsA before chilling (Figure 1D). Image analysis by image J showed a non- significant difference in mean grey values between control, chilling stressed plants, and plants pretreated with exogenous AsA (Figure 1E); however, the leaf mean integrated intensity normalized to the leaf area was significantly decreased from 1875.0 in untreated control plants to a level of 463.4 in chilling stressed plants and increased to 1372.9 in stressed plants pretreated with exogenous AsA (Figure 1F), reflecting that AsA treatment alleviated the chilling stress-induced damage in tomato plants.

### 2.2. Oxidative Damage

**Figure 2:**
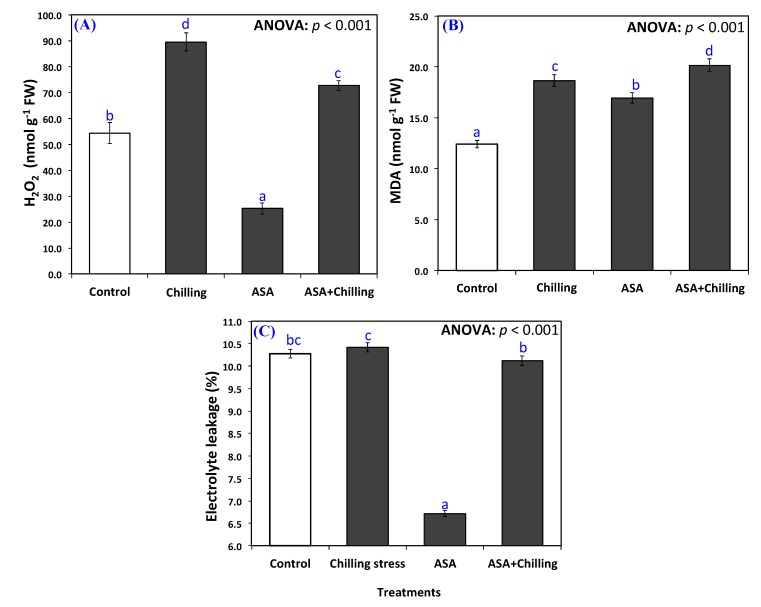
Oxidative damage of tomato plants with regards to (**A**) hydrogen peroxide, (**B**) lipid peroxidation (MDA) (**C**) electrolyte membrane leakage under chilling stress and pretreated with ascorbic acid. Data as mean and bars indicate the standard error for mean. Bars mentioned with various lowercase letters are significantly different in accordance with DMRTs.

Oxidative damage was determined by measuring the hydrogen peroxide and electrolyte leakage. Chilling induced oxidative stress significantly by triggering the generation of hydrogen peroxide, and AsA was effective in mitigating the oxidative damage to considerable levels (Figure 2). Untreated plants displayed a H_2_O_2_ of 54.33 nmol g^−1^ FW, which significantly and intensely amplified to a level of 89.56 nmol g^−1^ FW exposed to chilling stress concern (Figure 2A). The minimum hydrogen peroxide amount (25.20 nmol g^−1^ FW) has been reported in plants pre-treated with AsA; nevertheless, seedlings exposed to chilling stress and pre-treated with AsA revealed hydrogen peroxide amounts of 72.72 nmol g^−1^FW. Hence, the exogenous application of ascorbic acid decreased H_2_O_2_ accumulation significantly in plants under chilling stress (Figure 2A). Furthermore, membrane leakage always associated with plant responses to stresses. The Membrane leakage in tomato is at its least degrees in plants pre-treated with AsA (6.7%, Figure 2B). The electrolyte leakage in untreated control group and plants under chilling stress and treated with exogenous AsA was non- significantly different from each other (10.3% and 10.12% respectively) however, stressed plants recorded the maximum degree of electrolyte leakage of 14.4%. 

### 2.3. Antioxidant Capacity and Ascorbic Acid Content

**Figure 3:**
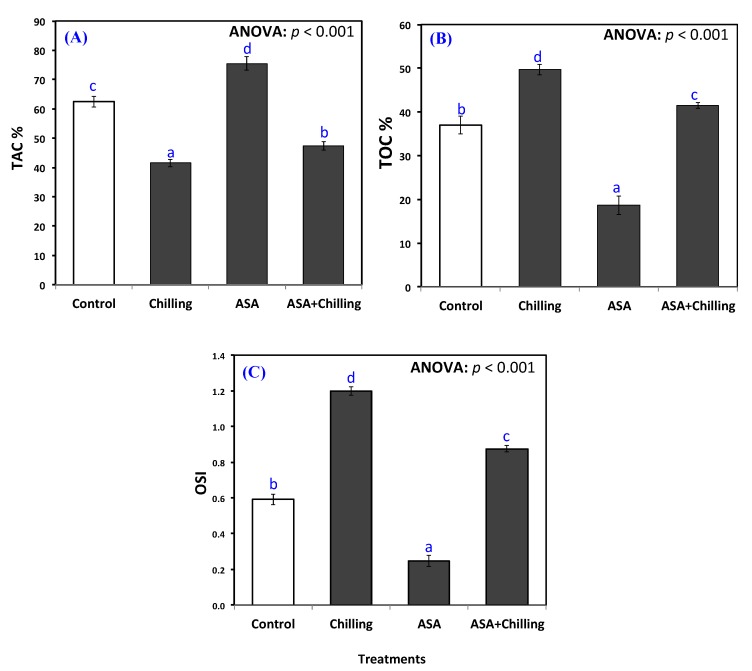
(**A**) Total antioxidant capacity (TAC), (**B**) total oxidative capacity (TOC), and (**C**) oxidative stress index (OSI) of tomato plants exposed to chilling stress and pretreated with ascorbic acid. Data as mean and bars indicate the standard error for mean. Bars indicated with various characters are significantly different in accordance with DMRTs.

The exogenous addition of AsA to plants increased total antioxidant capacity (TAC%) from 41.48% in chilling-stressed seedlings to a maximum of 75.5% (Figure 3A). Control plants and plants exposed to chilling with a previous treatment of AsA showed very similar results, however with significant difference between both, in favor of the latter (Figure 3A). 

Both total oxidant capacity (TOC, µmol g^−1^ FW) and oxidative stress Index (OSI) showed the same trend, with the plants exposed to chilling stress manifesting the highest values, the plants treated with exogenous AsA showing the least values and the control group along with the group of plants pretreated with AsA before chilling showing comparable results, although significantly different from each other (Figure 3B,C).

**Figure 4:**
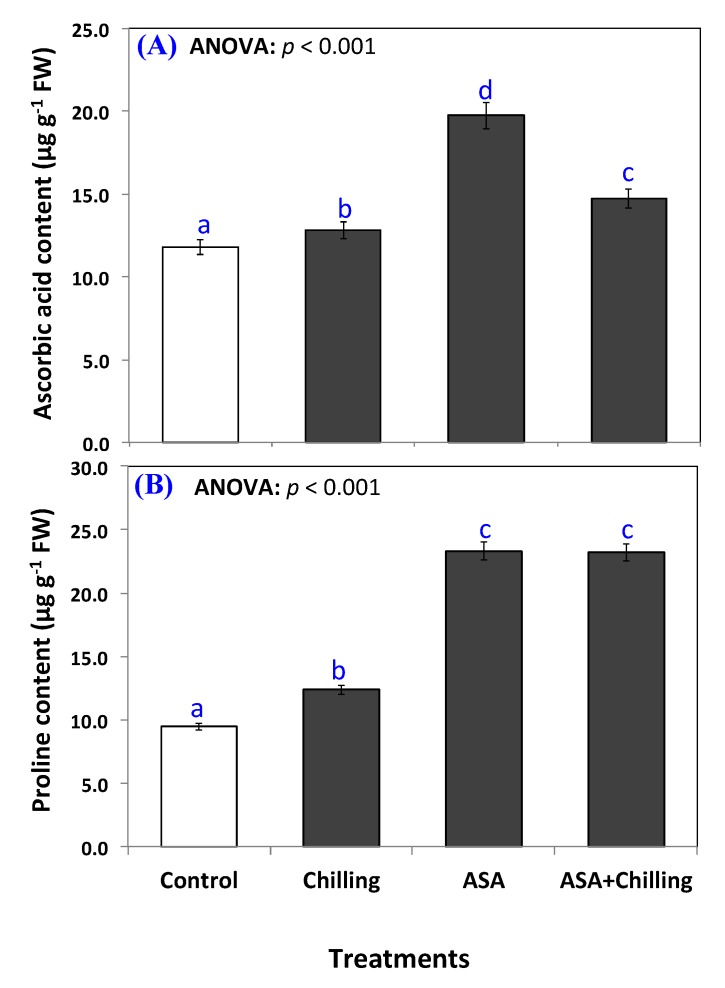
Ascorbic acid contents (**A**) and proline contents (**B**) in tomato seedlings untreated (control group) and treated with chilling stress and ascorbic acid. Data plotted as mean and bars represent the standard error for mean. Bars expressed with different lowercase letters are significantly different according to DMRTs.

Above all, incorporating AsA significantly (*p* < 0.05) elevated the cellular ascorbic acid level from 11.8 µg g^−1^ FW in control plants to 19.7 µg g^−1^ FW (Figure 4A). In addition, ascorbic acid significantly restored and improved the endogenous ascorbic acid in chilling-stressed seedling to an amount of 14.7 µg g^−1^ FW.

### 2.4. Proline Content

Proline increased from 9.5 µg g^−1^ FW to a level of 12.37 µg g^−1^ FW in chilling stressed plants (Figure 4B). AsA addition increased the proline content in plants significantly over the chilling-stressed group, although the level of increase was non-significantly different among both plants pretreated with AsA either exposed to chilling or not; 23.3 µg g^−1^ FW in AsA only treated plants and 23.2 µg g^−1^ FW in plants exposed to chilling stress after pretreatment.

### 2.5. Mineral Nutrition (NPK, Na, Mg)

**Figure 5:**
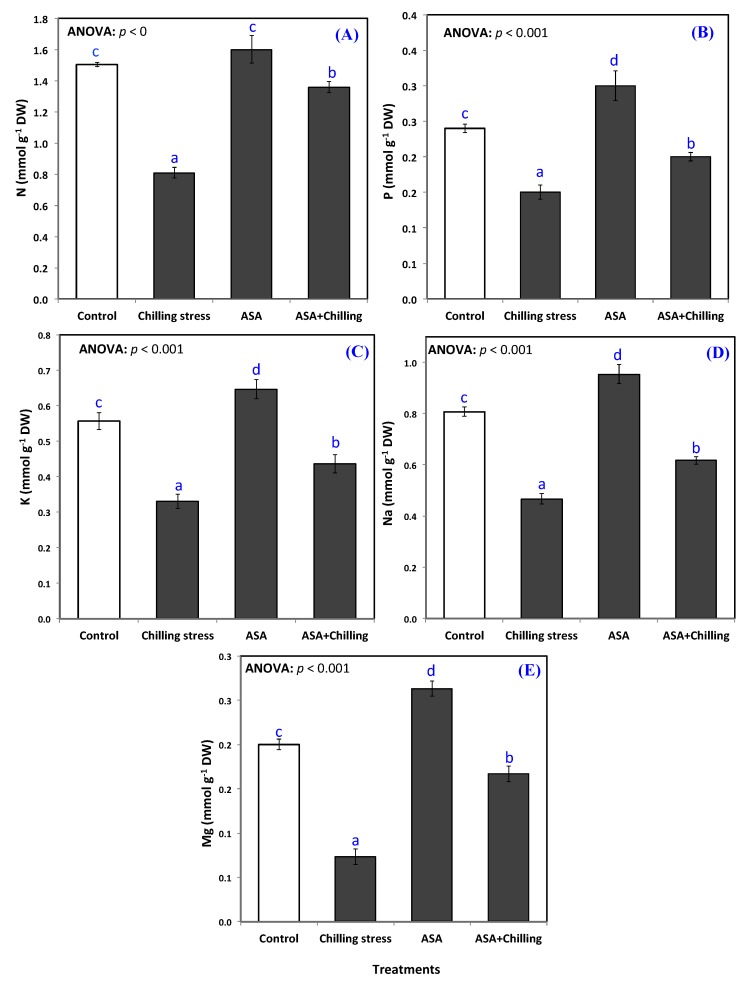
Mineral ions content (**A**) N, (**B**) P, (**C**) K, (**D**) Na, and (**E**) Mg in tomato seedlings untreated (control group) and treated with chilling stress and ascorbic acid. Data plotted as mean and bars represent the standard error for mean. Bars expressed with different lowercase letters are significantly different according to DMRTs.

Exogenous application of AsA substantially elevated the content of the plants of N, P, K to an amount greater than those of the controlled plants. Seedlings pretreated with exogenous AsA and exposed to chilling stress showed minerals contents higher than stressed ones (Figure 5A–E). In control plants, the content of minerals nutrients N, P, K, Na, Mg displayed an amount of 1.5, 0.24, 0.56, 0.8, and 0.2 mmole g^−1^ DW; respectively, while, AsA application significantly amplified minerals content (N, P, K, Na, Mg) in chilling-stressed seedlings to a level of 1.36, 0.20, 0.44, 0.62, and 0.17 mmole g^−1^ DW; respectively (Figure 5A–E). 

### 2.6. Photosynthetic Pigments (Chl-a, Chl-b) and Anthocyanins

Photosynthetic pigments are important stress markers. Certainly, the chlorophyll-a, -b contents revealed a higher-level following AsA application (Table 2). Chlorophyll-a displayed a level of 74.2, 14.6, 80.6, 26.54 µg g^−1^FW in the control plants, chilling-stressed, pre-treated AsA, chilling-stressed plants pre-treated with AsA, respectively. Chlorophyll-a injury was observed while comparing chilling-stressed seedling to control by the decrease in one-third of its content; nevertheless, AsA application restored the chlorophyll-a and chlorophyll-b level (Table 2).

Chl-b content was 6.8, 0.61, 7.2, and 1.13 µg g^−1^FW in the untreated control, chilling-stressed plants, exogenous AsA, chilling-stressed plants with exogenous AsA, respectively (Table 2). Chl-a, and Chl-b content exhibited marked destruction recognized from the minimization from 80.93 µg g−1 FW in control to 15.25 µg g^−1^ FW in chilling-stressed seedling. The photosynthetic content reported by ascorbic acid application, acquiring a level of 87.8 µg g^−1^ FW with a considerable improve greater than both control and chilling-stressed seedling (Table 2). The ascorbic acid application might eradicate the destruction of photosynthetic apparatus under acute chilling stress. 

Anthocyanins biosynthesis was severely affected by chilling shock as well. They increased from 0.6 Unit g^−1^FW in untreated control plants to a level of 1.08 Unit g^−1^FW in chilling-stressed plants. Application of AsA reserved and significantly improved the anthocyanin content from 1.08 to 1.23 in stressed plants pretreated with AsA (Table 2) revealed by one-way analysis of variance and DMRTs. 

### 2.7. Gene Expression of Heat Shock Protein and Antioxident Genes

**Figure 6:**
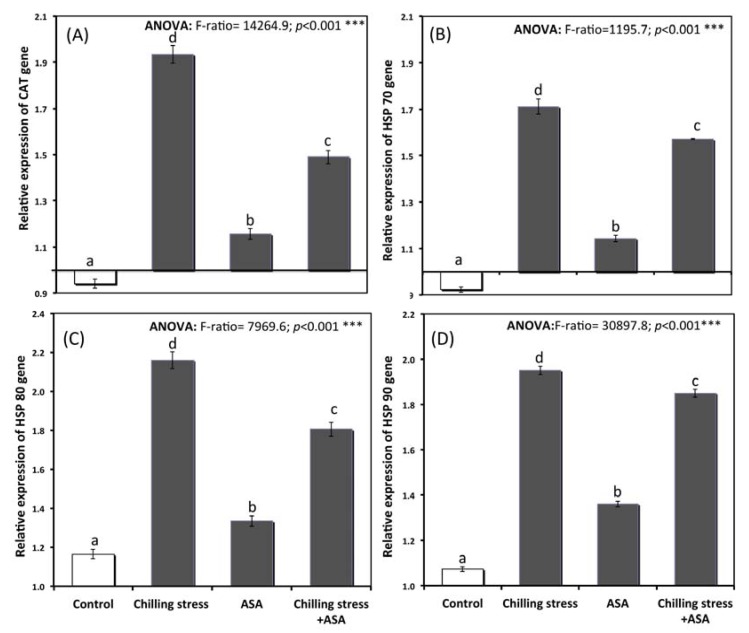
Relative expression of (**A**) CAT gene, (**B**) HSP70 gene, (**C**) HSPS 80 gene, and (**D**) HSP90 gene of tomato seedlings untreated (control group) and treated with chilling stress and ascorbic acid. Data plotted as mean relative gene expression and bars represent the standard error for mean. Bars expressed with different letters are significantly different according to DMRTs at *p* < 0.05.

The gene expression of heat shock protein and antioxidant genes involved in chilling stress response are given in Figure 6A–D. The expression level of catalase and heat shock proteins genes were augmented by 1.2–1.4-times over the control plants demonstrating that chilling stress- upregulating the expression of these genes. The pre-treated plant with 0.5 mM AsA showed a lower expression of HSP70, HSP80, HSP90, and CAT genes. This verified mitigation of chilling stress significantly in comparison to plants exposed to chilling alone. The relative expression of catalase gene (CAT gene) also increased significantly to 1.16, 1.93, and 1.49 in seedlings treated with exogenous AsA, chilling, and chilling stressed pretreated with AsA respectively (Figure 6A). Exogenous application of AsA successfully over-expressed cat gene, which increases the antioxidants capacity of tomato seedlings under chilling stress (Figure 6A). The heat shock protein genes were also significantly over-expressed in seedlings pretreated with exogenous AsA (Figure 6B–D). The heat shock proteins HSP70, HSP80 and HSP90 genes he catalase enzyme (1.6, 1.8, and 1.9 in chilling stressed plants pretreated with exogenous AsA; respectively (Figure 6B–D).

## 3. Discussion

Chilling has been proven to show negative effects on plant growth and reduce crop production. As a means to cope with these extreme temperatures, plants develop their precise regulating mechanisms and the use of chemical compounds known to reduce the harmful impact of severe temperature may be crucial [28]. Among the various strategies that have been used recently for the induction of tolerance to abiotic stress in plants is the seed and/or plant priming [9]. 

Negative effects of chilling on rice seedling growth were apparent in the form of reduced elongation of roots and shoots, as well as less biomass accumulation [9]. Under low temperature, disturbance in seedling morphology is a secondary expression of chilling-induced damage to cell organelles and its interference with key physiological processes [8]. Chilling stress might have reduced the growth of the seedling by suppressing cell elongation and division or/and metabolic imbalance in plant tissues [29].

### 3.1. Phenotypic Observations and Leaf Morphology

Various deformations in the leaves of tomato; that were exposed to chilling stress (4 °C for 7h), were observed (Figure 1, Table 2). These chilling-induced phenotypic results are inconsistent with that reported by Hussain et al. [9,30]. Chilling adversely affects the growth and development of plants by influencing water relation and mineral uptake [20]. Temperature extremes impede cellular growth and proliferation by restricting the cell cycle transition and influencing the functioning of key regulatory enzymes. Leaf disorders during cold exposure may also arise from the differences between water loss by transpiration and water root absorption and translocation. That could result from a lower hydraulic conductance in the root system, or root death, once the coffee root system is extremely sensitive to cold [31]. Similar to what was found by Alayafi [28], the AsA pretreated plants showed obvious minimization for stress, as confirmed through the preservation of plant leaf turgor and structure [32].

Chilling-induced limitation in water status disturbs the osmotic balance, impairs metabolic activity at the cellular level, and increased ROS affect DNA, RNA and protein structures, reduce respiration and ATP production thereby restricting seed germination and vigor [33]. However, AsA is known to regulate cell division and mediate cellular signaling for active elicitation of stress responses [21].

The effect of chilling stress on the growth and development of a plant is stage-dependent [34]. Most studies focus, therefore, on the germination and the emergence of seedlings, where chilling stress shows a negative seasonal impact on the crop quantity and quality [20]. Fewer studies on growth and development have been conducted under chilling stress using post-emergent seedlings stages [34] and the current study.

### 3.2. Oxidative Damage and Antioxidant Capacity

Oxidative damage was determined as a measurement of hydrogen peroxide and electrolyte leakage (Figure 2). Chilling potentially enhances the release of fatty acids by inducing galactolipase, up-regulating the activity of lipoxygenase, and free radical-mediated damaging effects in plants [35]. Xu et al. [36] found that priming improved the chilling tolerance in tobacco during seed germination and seedling growth by the activation of the antioxidant system in the plant tissues. In the current study, the results are consistent with those revealed by Kader et al. [29] who demonstrated increased oxidative damage in wheat due to excessive generation of hydrogen peroxide due to chilling stress. AsA proved effective in improving the TAC (increasing it by 2 folds and decreasing TOC to the half, minimizing the oxidative damage in the present study (Figure 3). The increased level of antioxidant capacity confers the ability of plants to scavenge ROS and to withstand the chilling stress [37]. Reduced oxidative damage in AsA treated seedlings may be due to the up-regulation of the antioxidant system reflecting in reduced accumulation of ROS also of its role as potent ROS scavenger and redox component [38].

An increased rate of solute leakage in tissues is often correlated with the appearance of chilling injury (CI) symptoms [39] and measurement of the injury severity in many crops [40]. We found that electrolyte leakage in stressed plants increased. This was also recorded by Saltveit [39] who showed an enhancement in electrolyte leakage from tomato pericarp discs chilled at 2.5 °C after 3 days. However, pericarp discs from the same cultivar harvested during winter exhibited an increase in electrolyte leakage after 6–7 days of chilling at 2.5 °C [41]. Some crops are exceptions and show no increases in electrolyte leakage as a result of chilling stress. Electrolyte leakage was reported to be unaffected by the chilling of peach [42]. In the AsA treated plants the leakage decreased and those pretreated with AsA before being exposed to chilling showed a very near value compared to the control group which assures the role of exogenous AsA in alleviating the effect of chilling. Husain et al. [9] also reported that seed priming treatments effectively alleviated the negative effects of chilling stress.

### 3.3. Ascorbic Acid and Proline Contents

Oxidative damage caused by chilling intensifies due to the disequilibrium between oxidants and antioxidants [32]. Plants have evolved antioxidant protection mechanisms like enzymatic antioxidant and non-enzymatic antioxidants to avoid or combat the harmful effects of ROS to mitigate such circumstances, and to safeguard the plants against oxidative stress. [43] and contribute significantly to the antioxidant potential of plants [44].

In the current study, the chilling stress increased both proline and endogenous AsA significantly compared to the control plants. The addition of exogenous AsA either alone or before the exposure to the chilling resulted in even higher contents of both (Figure 4). Cao et al. [45] reported that proline content in chickpea plants exposed to chilling stress increased compared to the control. The same observation was reported with Pepino [46], and Casava plants [47] when all were subjected to stresses.

Proline is an important amino acid protecting plants against stresses through its active involvement in osmoregulation and antioxidant molecule [48]. Proline mediates stress signaling, scavenges ROS and maintains osmolarity of cells under stressful conditions, and in the present study, AsA mediated enhancement in proline reduced the oxidative damage by strengthening antioxidant system [49]. Increased proline protects the photosystem II functioning by eliminating the ROS generated in chloroplast due to stress [50]. Exogenous AsA mediated improvement in proline accumulation under normal and chilling stress conditions may have resulted from its effects on its metabolizing pathways. Stress-mediated increase in proline results from enhanced synthesis and reduced catabolism of proline [8,51] and AsA application may have imparted differential regulation on proline accumulation. Similar to our results, Hussain et al. [30] have also demonstrated improved accumulation of proline and sugars in chilling stressed maize plants. Exogenous proline application prevents oxidative damage to cells by up-regulating the antioxidant system and in the present study increased proline accumulation due to AsA application confirms the probable role of AsA in proline mediated growth regulation under stress [52].

The increase of proline linked to stress might be because it is a compatible solute for sustaining the osmotic equilibrium enabling the plants to adjust to undesirable conditions [53]. Many studies have demonstrated that heat tolerance in tomatoes and Arabidopsis is supported by an accumulation of proline levels in leaves [54].

Plants exposed to chilling stress, increased of its endogenous ascorbic acid as a predominant stress response [29]. Similar to our findings in the current study, pre-treatment with 0.5 mM increased endogenous AsA significantly when compared with AsA-untreated stressed plants [47], since exogenously applied ascorbic acid influences the endogenous AsA content [50].

Ascorbic acid is a strong antioxidant and the level of stress tolerance is positively correlated with ascorbic acid content [55]. In the study by Kader et al. [29] performed with two types of wheat, it was shown that endogen ascorbate content increased due to chilling stress. Tambussi et al. [56] reported that application of L-galactono-1, 4 lactone (Gal), which is the precursor of ascorbic acid, before chilling stress increased the reduced ascorbate content in leaves and decreased the oxidized ascorbate content [46]. Improved development of AsA-treated plants might be resulting from mitigation of oxidative stress by antioxidant system developed by high-content of endogenous AsA, SOD and CAT obtained in this investigation which preserved photosynthetic pigments and reinforced higher relative water content [9].

The application of ascorbic acid, however, allows the plant to develop stress tolerance under different stress conditions [57]. The endogenous ascorbic acid content was found to be significantly higher than the control group particularly in plants which had been subjected to pre-treatment with ascorbic acid. The explanation for this rise in ascorbic acid may be the indication for plant stress tolerance [46]. As a consequence, exogenous use of ascorbic acid is likely to increase those endogenous parameters to control chilling stress effects.

### 3.4. Mineral Nutrition (NPK, Na, Mg)

Abiotic stresses hinder the uptake of nutrients by down-regulating the expression of transport proteins including NRT1, AMT1, and PMT1 [58]. Reports discussing the role of AsA in plant mineral uptake and assimilation are scanty. Temperature fluctuations in root zone adversely influence the uptake of crucial mineral ions, including N, P, and K [59]. Chilling, in the current study, has led to significant reduction in Na, Mg, N, P, and K contents in plant leaves (Figure 5). Ions, such as magnesium (Mg^2+^) and calcium (Ca^2+^) ions, play important roles under abiotic stress, which has received little attention. Magnesium ion has a key role in both protein synthesis and maintaining chlorophyll content [60], whereas calcium ion is an important signaling molecule elaborate in several biochemical processes and responses to both biotic and abiotic stresses [61].

However, this intake was alleviated by the addition of AsA. This alleviation effect of exogenous AsA was also reported by Alayafi, [28]; Athar et al. [62]. The mechanism of AsA-mediated ion homeostasis is still unclear, but is possibly due to the improvement of cell membrane stability and/or plasma membrane Na+ -H+ antiporter SOS1 [63]. Another possible explanation is that increased uptake of mineral elements like N, P, K and Mg in AsA treated plants may have directly affected the metabolism by regulating the key functions like enzyme activity, protein synthesis, chlorophyll synthesis and osmoregulation [64].

### 3.5. Photosynthetic Pigments (Chl-a, Chl-b) and Anthocyanins

Stresses significantly reduce the synthesis of photosynthetic pigments by reducing the uptake of Mg and improving the chlorophyllase activity in addition to the significant damage to chloroplast apparatus [65]. The inhibitory effect of chilling stress on plant growth have been ascribed to a reduction in photosynthetic capacity and carbon metabolism [66]. Chilling increases generation of ROS and reduces the enzymatic activities in chloroplast, and leads to accumulation of hydrogen peroxide resulting in declined altered photosynthesis [57,67]. In several studies, freezing temperatures have been shown to have a detrimental effect on photosynthesis [56]. Kaur et al. [68] reported a decline compared with the control of chlorophyll in chickpea-leaves exposed to cold stress. In maize plants subjected to chilling stress, has determined decreased chlorophyll and carotenoid levels [55]. Kalisz et al. [69] have also demonstrated a significant decline in chlorophyll and photosynthesis in basil exposed to chilling stress. In the current study, chilling stress affected the synthesis of Chl a and b significantly compared to the control plants, and addition of AsA as a pretreatment prevented the drastic effect (Table 2). The decrease in both Chl a and b could be explained as cold temperatures limit light energy photochemical use, leading to an excess of energy absorption, hence, many evergreen plants were found to lower their energy absorption in winter by decreasing Chl content and up-regulating photoprotective mechanisms [70]. The involvement of oxidative stress often accompanies PSI photoinactivation in vivo, e.g., due to an overreduction of the acceptor side of PSI linked to decreased CO_2_ carboxylation [70,71].

However exogenous AsA application has been reported to prevent photosynthetic arrest by improving the uptake of Mg, chlorophyll synthesis and the activity of Rubisco with a concomitant reduction in ROS generation and lipid peroxidation [57]. The present study advocates the beneficial effect of exogenous AsA in the protection of photosynthesis and growth through improved chlorophyll production. Pretreatement of plants exposed to chilling with AsA was reported to enhance the photosynthetic pigments in Casava [9], pepino [46] and tomato [28]. It was reported that the foliar spray of AsA enhanced chlorophyll a, b and carotenoids content in wheat and basil exposed to water stress [72].

Anthocyanins are water-soluble flavonoids, and their accumulation imparts photoprotection to plants under stressful conditions leading to maintenance of photosynthesis and other related plant functions [73]. Similar to our results, Ebrahimian and Bybordi [74] have demonstrated increased anthocyanin production in drought-stressed sunflower due to AsA treatment. In the present study also, AsA imparted significant enhancement in anthocyanin content probably preventing the chilling mediated deleterious effects on chloroplast structure and functioning, however exact mechanisms are not known. Kalisz et al. [69] have also demonstrated a significant decline in chlorophyll and photosynthesis in basil exposed to chilling stress.

### 3.6. Relative Gene Expression

Catalase is a crucial ROS scavenging enzyme leading to the elimination of hydrogen peroxide in the cell cytoplasm [8,75]. Increased antioxidant activities protect membrane structure and functioning hence leading to better plant performance [44]. Lee and Lee [76] have also demonstrated increased expression of antioxidant enzymes in cucumber exposed to chilling stress. CAT contributes to the scavenging of H_2_O_2_ [77]. Seed priming-induced increases CAT activities of rice seedlings have also been reported by Khaliq et al. [78] and Akram et al. [50], Ibrahim, and Opade [47].

HSPs are an essential group of proteins induced by environmental stress to protect plants from stress-mediated damage and to assist in repairing the damage caused by the stress [79,80]. HSPs are molecular chaperones involved in vivo enzyme and protein biogenesis. Increased expression of HSPs protects the structural stability of cold labile proteins [81], and AsA mediated enhancement in their expression indicates the potentiality to preserve protein structure and functioning.

Oxidative response also includes chilling stress-response genes [82]. Plant ROS levels also influence Hesp’s expression that responds to heat stress [83]. Both these results indicate a combination of ROS and temperature signals.

Plants Hsf and Hsp respond to stress under the thermal stress by changing their rates of transcription or the stability of their proteins [84]. Moreover, improvements in the AGO1 conformation, the elimination of passenger strands from the miRNA / miRNA* double chains complex and the formation of the RISC silence complex involve the connection of Hsp90 dimers during the miRNA biosynthesis cycle [85]. Such findings indicate that Hsp and Hsf may have a possible role in the resistance of heat stress and miRNA biosynthesis. Several studies have shown that miRNAs are able to control the HSF function by mediating the response to heat stress [86,87]. Although miR319 has recorded heat-induced expression in several species [88], it is still not understood how miR319 has been associated with heat stress control [89].

In the current study, the chilling stress resulted in enhanced relative expression of HSP70, HSP90, HSP80, and CAT genes. This was also observed by Alayafi [28] since Reactive oxygen species (ROS), which are regarded as stress signaling molecules [84], can be produced as a result of both chilling and heat stress [90]. Increased expression of HSPs observed in the present study may have also reduced the intensity of oxidative damage caused due to chilling, resulting in strengthening of tolerance to deleterious effects of stresses. Tomato seedlings over-expressing chloroplast HSP exhibit reduced the production of ROS and lipid peroxidation reflecting in increased photosynthetic performance [91].

AsA mediated enhancement in expression levels of HSPs under chilling stress confirms the existence of crosstalk link regulating the growth and development of tomato seedlings under chilling conditions. This was also reported as a result of seed priming by Hussain et al., [9] and Alayafi [28]. Pandey and his co-authors [92] found that membrane RNA helicases recognized, contributing to development, and stress tolerance. It is settled from the findings that AsA improved chilling stress tolerance of tomato seedlings concerning transcriptional induction of associated defensive genes, similar to the results of Kosová et al. [93] and Park and Park [94].

## 4. Conclusions

In conclusion, the present investigation revealed the significant potential role of AsA in alleviating the negative impacts of chilling stress in tomato plants by regulating antioxidant functioning, osmolyte, and ion homeostasis, and expression of catalase and heat shock proteins. Exogenous application prevented oxidative damage to chilled stressed tomato by up-regulating the antioxidant-functioning with a subsequent decline in the oxidant status. Ion homeostasis and increased chlorophyll and anthocyanin synthesis due to AsA treatment positively regulated the growth performance in tomato under chilling stress. Therefore, AsA application can be exploited to improve chilling tolerance in tomato by its active involvement in key regulatory functioning.

## 5. Materials and Methods

### 5.1. Experimental Design and Stress Condition

Tomato was grown in optimized soil for one month in a greenhouse of Botany Department, Faculty of Science, Suez Canal University, Egypt. The seedling was transferred and grown hydroponically in half-strength Hoagland nutrient solution [95]. The seedling was kept for one-week in the greenhouse at 60–70% relative humidity and temperature range 24–28 °C /20 °C day/night. The plants are divided into two groups; the first group is incubated in H_2_O. Whereas the second group is incubated in ascorbic acid solution (0.5 mM for 48 h). After that, the plants let to grow back again for 5 days in half-strength Hoagland solution. The first group is divided into the Control group (grown at room temperature), and chilling group (exposed to 4 °C for 7 h/day). The second group are divided into ascorbic acid group, where the plants roots are presubjected to 0.5 mM AsA and let to grow at room temperature, and ascorbic acid with chilling group, where the plants are presubjected to 0.5 mM AsA and were exposed to chilling stress (4 °C) for 7 h/day. A pilot experiment is being processed to determine the optimum and the best concentration of AsA for root priming and according to the previous experiment of other authors. Triplicate treatment was done for each treatment and three individual plants for each of the replicates. The leaves were collected for further laboratory examination in liquid nitrogen and stored at −80 °C.

### 5.2. Leaf Measurements and Image Analysis

To evaluate the performance of tomato seedlings under different proposed treatments, seedlings were photographed with a digital camera. Digital images were further processed and analyzed by ImageJ, a robust program that was developed at the National Institutes of Health, USA. Calibrated ImageJ was used to generate various leaf measurements, including leaf area, leaf length, leaf blade width [96,97].

### 5.3. Determination of Chlorophyll (a, b) and Anthocyanins Content

The Chlorophyll (a, b) was extracted by 80% (v/v) acetone and determined in accordance to Holder [98]. While anthocyanin was extracted in acidified methanol and quantified, according to Mancinelli [99].

### 5.4. Determination of Electrolyte Leakage Lipid Peroxidation, and Hydrogen Peroxide

Electrolyte leakage (EL) was assayed according to the method of Dionisio-Sese and Tobita [100]. The tissue is raised in bi-distilled water; then, conductivity was measured before and after boiling for taking the respective electrical conductivities. Lipid peroxidation was quantified by the estimation of malondialdehyde content, which was estimated spectrophotometrically using thiobarbituric acid assays [101].

For the determination of hydrogen peroxide (H_2_O_2_), leaves were homogenized in 0.1% trichloroacetic acid, and absorbance was measured at 390 nm, according to Sergiev et al. method [102].

### 5.5. Determination of Total Antioxidant Capacity, Total Oxidant Capacity, and Oxidative Stress Index

Assay of total antioxidant capacity (TAC) and total oxidant capacity (TOC) following Erel [103,104] method. The oxidative stress index (OSI) is calculated as follow:

OSI (arbitrary unit) = TOC (μmol H_2_O_2_Eq/L)/ TAC (μmol Trolox Eq/L) (1)

### 5.6. Determination of Ascorbic Acid and Proline

Ascorbic acid was determined according to the method of Nweze et al. [105]. 25 mL of prepared juice was taken in each of six 100 mL conical flasks. 10 mL of 1 M H_2_SO_4_ was added and titrated with a standard iodine solution using 2 mL starch as indicator till the appearance of blue colour and then the amount of ascorbic acid was calculated. Proline was assayed by homogenizing leaves in 3% sulphosalicylic acid in accordance to Bates et al. [106].

### 5.7. Mineral Ions Content (N, P, K, Na, and Mg)

Total Nitrogen is being quantified according to according to Lennox and Flanagan [107] by Kjeldahl method. While the potassium and sodium quantification was achieved by Flame Atomic Absorption Spectrometry (Agilent, Hachioji, Japan). Phosphorus estimation in leaves was measured in accordance with Fogg and Wilkinson [108]. Finally, magnesium and calcium contents were quantified, according to Rowell [109] by atomic absorption spectrophotometer (AAS-Hitachi, Tokyo, Japan).

### 5.8. RNA Extraction and Real-Time PCR Analysis

Total RNA extraction kit (Sigma-Aldrich, Munich, Germany) is used for the isolation of RNA from tomato leaves. Then the SuperScript cDNA synthesis kit is used for cDNAs formation. 18s gene was used as a reference gene in the relative expression of gene expression; primers sequence of catalase and heat shock proteins are in Table 1. The reaction is made of 2 µL of forward primer, 2 µL of reverse primer, 10 µL of SYBR Green Master Mix, 2 µL of the template, and sterile water for a total volume of 20 µL. Livak equation 2^−ΔΔCt^ are used for the calculation of relative gene expression [110].

### 5.9. Data Analyses

The Microsoft Excel 2016 and SPSS (version 23) are being used for biostatistics test, presenting and drawing data results. Inferential statistics for assessing and linking treatments by using one-way analysis of variance (ANOVA) and Duncan multiple range tests.

## Figures and Tables

**Table 1:** Shoot, root, and plant lengths and fresh of Tomato before exposure to chilling stress. Data represented as a mean of three replicate plants + standard error. Means with different letters are significantly different, according to Duncan’s multiple range tests (DMRTs).

Variable	Length (cm) Mean ± SE	FW (g plant−1) ) Mean ± SE
Shoot system	12.86 ^a^ ± 0.40	3.57 ^b^ ± 0.16
Root system	11.81 ^a^ ± 0.39	1.32 ^a^ ± 0.09
Whole Plant	24.67 ^b^ ± 0.40	4.90 ^c^ ± 0.24
**ANOVA**		
F-ratio	331.712	106.503
*p*-value	< 0.001	< 0.001

**Table 2:** Chlorophyll a, chlorophyll-b, and anthocyanin in tomato seedlings under different treatments (control, chilling stressed, AsA, chilling, and AsA). Data represented as a mean of three replicate plants ± standard error. Means with different letters are significantly different, according to Duncan’s multiple range tests (DMRTs).

Treatments	Chlorophyll-a	Chlorophyll-b	Anthocyanin
	µg g^−1^ FW	µg g^−1^ FW	Unit g^−1^ FW
**Control**	74.17 ± 1.00 ^a^	6.76 ± 0.03 ^a^	0.59 ± 0.01 ^a^
**Chilling stress**	14.64 ± 0.03 ^b^	0.61 ± 0.02 ^b^	1.08 ± 0.03 ^b^
**AsA**	80.61 ± 0.73 ^d^	7.17 ± 0.07 ^d^	0.68 ± 0.01 ^d^
**AsA + Chilling Stress**	26.54 ± 0.03 ^c^	1.13 ± 0.14 ^c^	1.23 ± 0.02 ^c^
**One-way ANOVA**			
F-ratio	2893.17	1947.63	249.58
*p*-value	*p* < 0.001	*p* < 0.001	*p* < 0.001
